# Clinical application of accelerated rehabilitation surgery in elderly patients with colorectal cancer

**DOI:** 10.1097/MD.0000000000022503

**Published:** 2020-10-09

**Authors:** Linxia Xu, Xianrong Li, Meixuan Song, Liang Xu, Xunlian Wu

**Affiliations:** Department of General Surgery (Gastrointestinal Surgery), Hospital of Southwest Medical University, Jiangyang District, Luzhou City, Sichuan Province, China.

**Keywords:** clinical outcomes, colorectal cancer surgery, elderly patients, enhanced recovery after surgery, routine care

## Abstract

**Background:**

Gastrointestinal malignant tumors are the most common malignant tumors in elderly people in China, resulting in an increasing trend of morbidity and mortality. We conducted a non-randomized controlled trial to compare the effect of enhanced recovery after surgery (ERAS) versus Routine care on clinical outcomes in elderly patients after colorectal cancer surgery.

**Methods:**

This is a single center, non-random, parallel-controlled clinical trial, 60 patients aged ≥65 years will be randomized for Case group ERAS and Control group (routine care).

**Results:**

This study will help to evaluate the clinical feasibility, safety and effectiveness of ERAS in elderly patients undergoing colorectal resection compared with routine care.

**Protocol registration number:**

ChiCTR2000034984

## 1 Introduction

The incidence rate and mortality rate of colorectal cancer in China are increasing year by year. Affected by diet and environment, the number of patients with rectal cancer has been high in China so that a large number of surgical treatments in hospital every year. Besides, the disease is also the main cause of death and a major public health problem as the number of patients is the second only to lung cancer and gastric cancer in China. It was estimated that 191,000 people died in 2015.^[1,2]^

With the improvement of living standard, the proportion of the elderly over 65 years old is increasing. Gastrointestinal cancer is the most common malignant tumor in the elderly over 75 years old in China, according to statistics, the annual incidence of colorectal cancer in the elderly over 75 years old in China is about 78,200. At the same time, with the progress of anesthesia technology, the number of elderly people who can receive surgical treatment has increased significantly.^[3,4]^ However, elderly patients with colorectal cancer after surgery are more likely to have adverse postoperative consequences, such as longer hospital stay, the occurrence of various complications, high readmission rate, etc, and the economic costs will be correspondingly high.^[5,6]^ Consequently, we present this research to prove the use of enhanced post-operative recovery (ERAS) which could reduce the adverse consequences of elderly patients after surgery, and improve the rehabilitation effect compared with routine care.

The surgical team has developed and implemented multidisciplinary care programs, included ERAS, which improves patient outcomes and reduces hospital stay.^[6,7]^ ERAS includes evidence-based changes in traditional care, such as consultation before admission, avoiding mechanical bowel preparation, shortening fasting, carbohydrate drinks for 2 hours before surgery, avoiding fluid overload, minimally invasive surgery, avoiding early removal of drainage tubes, and catheters, and multimodality of opioid retention analgesia, early feeding and activities. In 1 meta-analysis, ERAS during colorectal surgery was significantly associated with the reduction of postoperative complications, total hospital costs, and early recovery of gastrointestinal function, without increasing 30 day readmission or mortality.^[8]^ Another study showed satisfactory short-term clinical results and demonstrated that laparoscopic D2 lymphadenectomy combined with era is safe and feasible.^[7]^ ERAS is widely accepted to be the gold standard for the treatment of colorectal disease in patients.^[8,9]^ However, there are few articles on the elderly. Our study intends to analyze the effectiveness and safety of clinical application of ERAS in elderly patients with colorectal cancer, and to observe its impact on postoperative recovery of elderly patients with colorectal cancer.

## 2 Methods

### 2.1 Trial design

**Figure 1:**
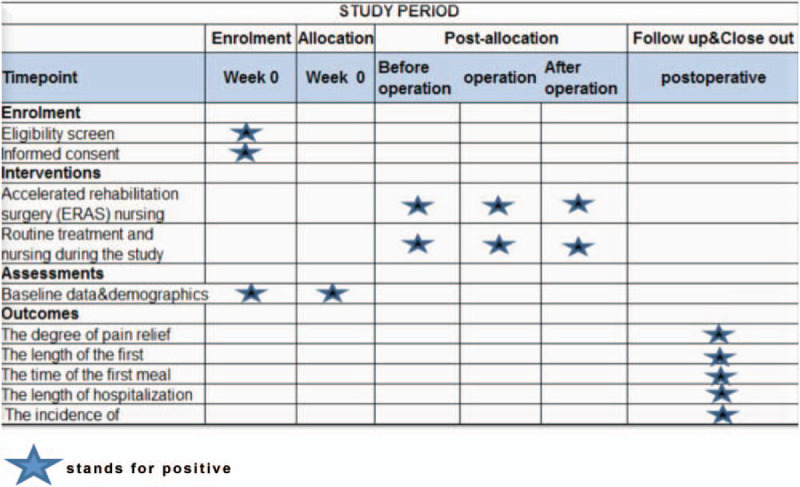
Study period (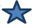 stands for positive).

This will be a non-random control trail with single center experience. Elderly patients will be included from the Affiliated Hospital of Southwest Medical University. We developed this protocol based on the 2013 statement of standardized protocol interventions: recommendations for intervention trials (SPIRIT).^[10]^Figure 1 shows the schedule for registration, assessment, intervention, and follow-up in accordance with spirit guidelines.

### 2.2 Patient population and eligibility criteria

**Figure 2:**
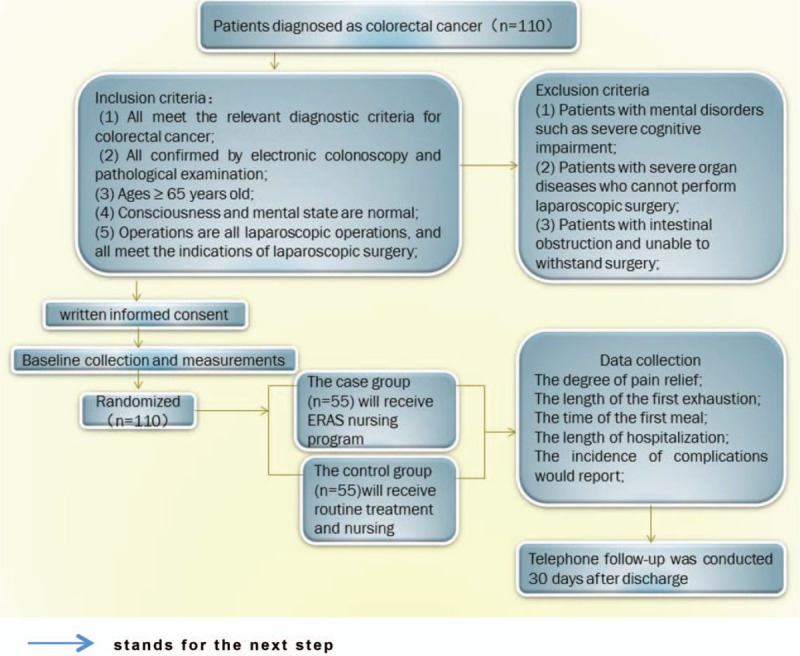
Flow chart (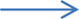 stands for the next step).

Inclusion criteria:

(1) all meet the relevant diagnostic criteria for colorectal cancer;
(2) all confirmed by electronic colonoscopy and pathological examination;
(3) age ≥ 65 years old;
(4) consciousness and mental state are normal;
(5) operations are all laparoscopic, and all meet the indications of laparoscopic surgery;

Exclusion criteria:

(1) patients with mental disorders such as severe cognitive impairment;
(2) patients with severe organ diseases who cannot perform laparoscopic surgery;
(3) patients with intestinal obstruction and unable to withstand surgery;

Flow chart is shown in Figure 2.

### 2.3 Intervention

The case group will receive ERAS:

a. the first guidance: to explain the disease knowledge of patients, simply understand the stoma care methods, without detailed guidance of stoma care methods;
b. preoperative preparation: no intestinal preparation was needed;
c. intraoperative intervention: the abdominal cavity was washed with warm water to avoid hypothermia
d. follow up guidance: the intervention staff only need to conduct telephone follow-up 1 month after discharge to understand the status of patients.

The control group will receive routine treatment and nursing.

a. First guidance: nurses face the patients on admission, one-to-one through oral and written guidance
  The patients understood the disease-related knowledge, stoma nursing methods, and matters needing attention in daily life;
b. Preoperative preparation: according to clinical routine operation, assist patients to improve intestinal preparation;
c. Intraoperative intervention: according to the traditional process, there is no need for heating intervention and other measures;
d. follow up guidance: the intervention personnel kept in touch with the research subjects during the trial, and 1 month after discharge. Telephone follow-up was conducted to understand the nursing ability and quality of life of patients.

### 2.4 Data collection

We will collect the relevant indicators based on the final outcomes. The research assistant will collect the following data:

Basic information:

Age, gender, education level, body mass index, pathological type, and stage of cancer, ASA score of anesthesia, preoperative albumin, and globulin level.

Stress response indicators: Fasting blood glucose, C-reactive protein (CRP)

Immune function index: T lymphocyte subsets (CD3 +, CD4 +, CD8 +, CD4 + / CD8 +) and immunoglobulin (IgG, IgM, IGA)

Psychological assessment: Zung's self-rating Anxiety Scale (SAS) was used to evaluate the anxiety of the 2 groups

Postoperative recovery index and Economics: Anal exhaust time, first fluid intake time, total hospitalization time, total hospitalization expenses, gastric tube indwelling time and urinary catheter indwelling time

Postoperative complications: Anastomotic leakage, intestinal obstruction, pulmonary infection, urinary tract infection, urinary retention, wound infection.

### 2.5 Laboratory analyses

The preoperative values of laboratory tests (BUN, CREA for renal function; AST, ALT for liver function, CRP, CK, CK MB, BNP for Cardiac function; HB, WBC, PLT, PaO2/FiO2, and PaCO2 for blood routine) in the last 7 days before operation were analyzed as baseline values (preoperative). Extra blood samples will be collected on day 1 after surgery for laboratory evaluation. Postoperative complications were evaluated by a doctor certified by an independent committee. In this trial, he will be guided on the diagnostic criteria for specific postoperative complications. The specialist will evaluate all participants until 30 days after surgery.

### 2.6 Postoperative complications

A medical specialist, the outcome assessor, will be instructed about diagnostic criteria in this study for specific postoperative complications. All participants will be evaluated by the outcome assessor until 30 days after surgery. And we will use evidence-based process of comprehensive geriatric assessment (CGA)^[11]^ to evaluate an older patient's general condition and the risk of adverse outcomes

### 2.7 Study outcomes

The degree of pain relief, the length of the first exhaustion, the time of the first meal, the length of hospitalization, and the incidence of complications would report.

### 2.8 Adverse events

The most common adverse events in patients who died following a cancer resection were related to the surgical care, medical care, critical care, and staffing, communication and documentation.^[12]^ We will train the research personnel, implement the research plan strictly according to relevant regulations, and guarantee the quality of data collection.

### 2.9 Withdrawal and dropout

The trial will be concluded at any stage when the main investigator considers that there is an unacceptable risk of serious adverse events, or the patient refused to continue, withdrew the consent, and violated the inclusion or exclusion criteria or trial protocol. Interim analysis will not be conducted; the patient's physical condition will be paid attention after recruitment. However, the hospital will develop the final criteria for early termination of the study, which may be based on clear evidence that one of the trial groups deteriorated, that there was no significant benefit of reasonable doubt in the measurement of the main outcome, as well as the existing literature and clinical understanding of the disease.

### 2.10 Confidentiality

On recruitment, the researchers gave each participant an unique number, and only the number is used to identify participants. The information sheets and electronic documents collected will be used for security offices locked in any safe. The recipient list and notification approval form will be securely identified. All digital files will be password protected and stored securely. The trial sponsor has access to the final trial dataset.

### 2.11 Statistical analysis

In this study, the sample size was estimated by comparing 2 independent samples. Considering the loss of follow-up, the sample size was increased by 10%, so the final sample size (N1 + N2) ∗ (1 + 10%) was 110. Therefore, 55 patients in the case group and 55 patients in the control were selected in this study. SPSS 22.0 statistical software was used for data entry and statistical analysis (*P* < .05 was statistically significant). Statistical description was used for general data, ANOVA of repeated measurement was used for quantitative data, and rank sum test was used for rank data.

### 2.12 Trial oversight

The trial management team, including the lead investigator, test coordinator, test manager, data manager, and test statistician, maintains weekly contact. A meeting will be set by the Committee (TSC) at least once a month to check the progress of the trial and ensure that it is conducted in accordance with the protocol, relevant regulations and good clinical practice principles. In addition, a professional team will be set up to review the test schedule and safety data. The team will be independent of the experimental researchers and consists of 3 independent members, including 2 clinical experts and 1 trial statistician.

### 2.13 Dissemination policy

Patient public participation members will play a key role in our findings. The results will be disseminated to patients, health professionals, committee members, policy makers, and the public widely. And the study will be published in Medicine. Conclusion strengthening postoperative recovery is an improved process of evidence-based nursing for surgical patients.

## 3 Discussion

ERAS is a process of improving the condition of surgical patients based on evidence-based nursing. The implementation of strengthening postoperative recovery can greatly improve the clinical effect and reduce the cost, making eras an important standard of surgical value-based nursing.^[13]^ An article summarizes the ERAS results of elderly patients (≥65 years) after colorectal surgery. The median overall incidence rate is 23.5%, the median postoperative loss is 6 days, and the overall postoperative complication rate quite high. Conducting this review shows that it is challenging to compare postoperative complications between studies, and the exclusion criteria are different, because there is no uniform way to define or evaluate complications. Many studies only evaluate selected complications or range of complications by organ system. Patients receiving ERAS require lower upfront costs than traditional caregivers. However, the evaluation results of this study should be based on the long-term results and functional results of patient treatment, so the overall cost-effectiveness has not been fully determined, and longer follow-up data is required for further research. This review compare ERAS with routine care, in which primary outcomes including the degree of pain relief, the length of the first exhaustion, the time of the first meal, Length of stay, and the incidence of complications would report. In this study, we want to evaluate the clinical effect of ERAS and traditional care in elderly patients with colorectal cancer surgery through retrospective analysis of the results and risk factors for postoperative complications.

## Author contributions

**Data curation:** Linxia Xu, Xunlian Wu.

**Formal analysis:** Meixuan Song.

**Investigation:** Liang Xu.

**Writing – review & editing:** Xianrong Li.

## Abbreviations

Abbreviation: ERAS = enhanced recovery after surgery.
